# Structural and physicochemical properties of bracken fern (*Pteridium aquilinum*) starch

**DOI:** 10.3389/fnut.2023.1201357

**Published:** 2023-06-20

**Authors:** Kehu Li, Tongze Zhang, Huanhuan Ren, Wei Zhao, Siqi Hong, Yongyi Ge, Xiaoqiong Li, Harold Corke

**Affiliations:** ^1^Key Laboratory of Plant Resource Conservation and Germplasm Innovation in Mountainous Region (Ministry of Education), Collaborative Innovation Center for Mountain Ecology & Agro-Bioengineering (CICMEAB), Institute of Agro-Bioengineering, College of Life Sciences, Guizhou University, Guiyang, Guizhou, China; ^2^Biotechnology and Food Engineering Program, Guangdong Technion-Israel Institute of Technology, Shantou, China; ^3^State Key Laboratory for Managing Biotic and Chemical Threats to the Quality and Safety of Agro-Products, Institute of Food Sciences, Zhejiang Academy of Agricultural Sciences, Hangzhou, China; ^4^Faculty of Biotechnology and Food Engineering, Technion–Israel Institute of Technology, Haifa, Israel

**Keywords:** bracken fern, starch, physicochemical properties, amylopectin structure, food bioresources

## Abstract

**Introduction:**

Bracken fern (Pteridium aquilinum) starch is a non-mainstream, litter-researched starch, thus the starch characteristics remain largely unknown.

**Methods:**

The structural and physicochemical properties of two bracken starches were systematically investigated, by use of various techniques that routinely applied in starch analysis.

**Results and Discussion:**

The starches had amylose contents of 22.6 and 24.7%, respectively. The starch granules possessed C-type polymorph with D (4,3) ranging from 18.6 to 24.5 μm. During gelatinization event, the bracken starches showed lower viscosity than typical for rice starch, and lower gelatinization temperature than typical for cereal starches. After gelatinization event, bracken starches formed much softer and sticky gel than rice and potato starch. The molecular weight and branching degree (indexed by Mw, Mn and Rz values) of bracken starches were much higher than starches of many other sources. The branch chain length distributions showed that the bracken starches were structurally similar to some rice varieties (e. g. BP033, Beihan 1#), as reflected by proportions of A, B1, B2, and B3 chains. Notable differences in some starch traits between the two bracken starches were recorded, e. g. amylose content, gel hardness, gelatinization temperature and traits of structural properties. This study provides useful information on the utilization of bracken starch in both food and non-food industries.

## 1. Introduction

Bracken fern (*Pteridium aquilinum*) is widely distributed in South and Central America, Oceania and Southeast Asia (1). There is a long history of human consumption of bracken fern as a major dietary source to obtain starch and fiber (2). Nowadays, bracken fern is not only an important ornamental plant which is widely used in garden landscaping, but also still serves as a food material in many countries. In Japan and China, the tender bud of bracken fern cooked as vegetable is a popular delicacy in Spring (3). In Southwest China, a kind of sticky cake made from bracken starch, namely ‘Jue Ba’, is a traditional and popular local food. ‘Jue Ba’ is usually cooked with cured pork, or directly eaten as a snack after toasted.

Despite its utilization as human food, bracken fern still remains to be a wild plant, there is no documentation regarding its cultivation yet. Studies on bracken fern are limited, and most of literature focused on the bracken toxins (1). Investigations into the nutrients of bracken fern are few. Previously, it is reported that bracken fern contains various nutrients, such like protein, carbohydrates, fat, vitamins, trace minerals and carotenoids (4). It was found that abundant starch granules accumulate in parenchymal cells of bracken fern rhizome (5), an up to 45.29% total starch content was reported for bracken fern (6). However, the characteristics of starch from this botanical source remain largely unknown. Some properties including morphological, crystal, pasting and *in vitro* digestion properties had been investigated, using only one sample obtained in China (5), but the molecular structure and many important physicochemical properties still remain undetermined.

There are mainly two types of polysaccharides in starch: amylose and amylopectin. Amylose is mostly linear with few branches while amylopectin is highly branched. Many factors can affect starch physicochemical properties, including the shape and size of starch granules, amylose/amylopectin ratio, and fine structure of amylopectin (7, 8). Fully knowing the physicochemical and structural characteristics of a starch is a prerequisite for further developing its utilization in food and non-food industries. For example, crop grains with lower amylose content are usually favored in fermentation to produce vinegar and white wine, and the strength of the starch-based film is largely determined by the molecular weight of amylose and amylopectin. In order to accurately describe starch quality, several indexes have been established and routinely used in main-stream starches, i.e., apparent amylose content (AAC), Rapid Visco Analyzer (RVA) pasting viscosity, gel consistency (GC), textural properties and thermal properties (9).

In this study, starch samples were extracted from the rhizome of two bracken ferns, which were grown in different environments. Then, the structure and physicochemical properties of these two starch samples were systematically investigated. The aim of this study is to broaden our knowledge on bracken starch characteristics, in order to provide a guideline for later exploring its potential utilization in food and non-food industry.

## 2. Materials and methods

### 2.1. Materials

Two bracken starch samples were used in this study. One (J_1_) was extracted from the rhizome of bracken fern grown in Yongxing City, Hunan province, China (113°E, 26°N) and the other (J_2_) was extracted from the rhizome of bracken fern grown in Huaihua City, Hunan province, China (109°E, 27°N). Both starch samples were extracted by the suppliers and sold online. J_1_ was purchased from online shop“Yongxing Lao Jia Tu Te Chan” and J_2_ was bought from “Xun Wei You Pin Te Chan Dian.”

### 2.2. Scanning electron microscopy

The starch granules were gold coated, then an SEM (Scanning Electron Microscope VEGA3, TESCAN, Brno, Czech Republic) was used to observe the morphology of the starch granules, with a voltage of 3.00 k V and a magnification of 1.50 k X.

### 2.3. Apparent amylose content

Apparent amylose content was measured according to the previously reported method (9). In brief, 100 mg starch sample was weighed into a 100 mL measuring flask, then dispersed with 1 mL of 95% ethanol. Next, 9 mL of NaOH (1 mol/L) was added, and the starch solution was gently shaken to avoid major lumps. The solution was boiled till completely clear. After cooling, the solution was diluted to 100 mL with distilled water. Finally, 500 μL of sample solution was well mixed with 200 μL of I_2_-KI (0.2–2%), 100 μL acetic acid (1 mol/L) and 9 mL distilled water in a 15 mL centrifugal tube. This final mixture was kept at room temperature for 10 min before measured by an spectrometer (UV 1800PC, Jinghua Instruments, Shanghai, China), at a wavelength of 620 nm. A standard curve was made simultaneously by using standard amylose (A0512, Sigma-Aldrich, Shanghai, China) and amylopectin (10,120, Sigma-Aldrich, Shanghai, China).

### 2.4. Particle size distribution

A laser diffraction particle size analyzer (S3500, Microtrac, Montgomeryville, PA, United States) was used to determine the particle size distribution of the starch samples, according to the manufacturer’s protocol.

### 2.5. Crystal properties

An X-ray diffractometer (3 kW/*D8 ADVANCE Da Vinci, Bruker, Karlsruhe, Germany) was used to reveal the diffraction patterns of starch samples. An X-ray tube Cu-Kα (nickel filter) at 40 kV and 40 mA was used as the X-ray source. The angular range of the diffraction angle (2θ) was 5°-40° with a step interval of 0.02°.

### 2.6. Pasting properties

A rapid visco analyzer (RVA4500, Perten Instruments, Hägersten, Sweden) equipped with Thermocline for Windows software was used to measure pasting properties. In brief, starch sample (3 g, 12% moisture basis) was mixed with 25 mL distilled water in a RVA sample can. The starch solution was heated following a temperature program: held at 50°C for 1 min, heated to 95°C in 3.8 min, and kept at 95°C for 10 min, cooling down to 50°C in 3.8 min and maintained at 50°C for 2 min. The peak time (Ptime), pasting temperature (PT), peak viscosity (PV), hot paste viscosity (HPV), cool paste viscosity (CPV) and the derivative parameters breakdown viscosity (BD = PV − HPV), setback viscosity (SB = CPV− PV), consistency (CS = CPV− HPV) were recorded or calculated.

### 2.7. Textural properties

The starch gel formed after RVA test was sealed in the sample can by Parafilm™ and kept at 4°C for 24 h. Then, a TA-XT2i Texture Analyzer (Stable Micro Systems, Godalming, United Kingdom) equipped with Texture Expert software (version 2.1) was used to measure the textural properties. The gel samples were pressed twice by a flat-ended, 5 mm diametered cylindrical probe for 10 mm distance at a speed of 1.0 mm/s. Hardness (HD), adhesiveness (ADH) and cohesiveness (COH) were recorded by the software.

### 2.8. Thermal properties

Thermal properties were measured by differential scanning calorimetry (Discovery DSC 25, TA Instruments, New Castle, DE, United States). In brief, starch sample (2 mg, dry basis) was mixed with 6 μL distilled water in an aluminum sample pan. The sample pan was hermetically sealed and equilibrated at room temperature for at least 2 h, before being heated from 30°C to 110°C at a rate of 10°C/min. An empty sealed pan was used as a reference. Onset (T_o_), peak (T_p_), conclusion (T_c_) temperatures and enthalpy of gelatinization (ΔH_g_) were calculated using the Universal Analysis Program, Version 1.9D (TA Instruments).

### 2.9. Fourier transform infrared (FTIR) spectrum analysis

A Nicolet iZ-10 FTIR instrument (Thermo Fisher Scientific, Waltham, MA, United States) was used to scan starch samples for fourier transform infrared spectra. Starch sample (5 mg) was mixed with 250 mg potassium bromide and pressed into film-coated tablet. The potassium bromide was considered as the background of the tablet. Wavenumbers from 400 to 4,000 cm^−1^ were measured at 4 cm^−1^ spectral resolution over 32 scans. The molecular structure of starch was calculated based on the absorbance ratio of 1047/1022 cm^−1^, 1,022/995 cm^−1^.

### 2.10. Branch chain-length distribution

The branch chain-length distribution of amylopectin was analyzed by high-performance anion-exchange chromatography (HPAEC) on a CarboPac PA-100 anion-exchange column (4.0*250 mm, Dionex) using a pulsed amperometric detector (PAD, Dionex ICS 5000 system). Data were acquired on the ICS5000 (Thermo Fisher Scientific, Waltham, MA, United States), and processed using chromeleon 7.2 CDS (Thermo Fisher Scientific, Waltham, MA, United States).

### 2.11. Molecular weight distribution analysis

Starch molecular weight was measured by a gel permeation chromatography-refractive index-multiangle laser light scattering detector (GPC-RI-MALLS). The differential refractive index detector (Optilab T-rEX, Wyatt Technology Co., Santa Barbara, CA, United States) was equipped with two tandem columns (300 × 8 mm, Shodex OH-pak SB-805 and 803, Showa Denko K.K., Tokyo, Japan). The data were acquired and processed using ASTRA6.1 software (Wyatt, Santa Barbara, CA, United States).

### 2.12. Average degree of branching

Average degree of branching was determined using a Bruker BioSpin GmbH NMR spectrometer equipped with a tempering unit. Starch sample (10 mg) was mixed with 1 mL of deuterated dimethyl sulfoxide-d6 (DMSO-d6) and fully dissolved at 80°C overnight. The mixture was centrifuged at 12,000 rpm and the supernatant was taken and pipetted into an NMR tube. The ^1^H NMR scanning was performed 32 times, at a Larmor frequency of 500.23 MHz. Data were collected and analyzed with MestReNova. Average DB was calculated with Equation: DB (%) = (I-1,6) / (I-1,6 + I-1,4)*100, where I-1,6 is the integrated signal at 4.77 ppm and I-1,4 is the integrated signal at 5.12 ppm, respectively.

### 2.13. Statistical analysis

All traits were measured in at least duplicate. Data analysis was performed using SPSS 25.0 statistical software program. Significance of differences between the means were calculated by the analysis of independent t-test (*p* < 0.05).

## 3. Results and discussion

### 3.1. Morphology of starch granules

**Figure 1:**
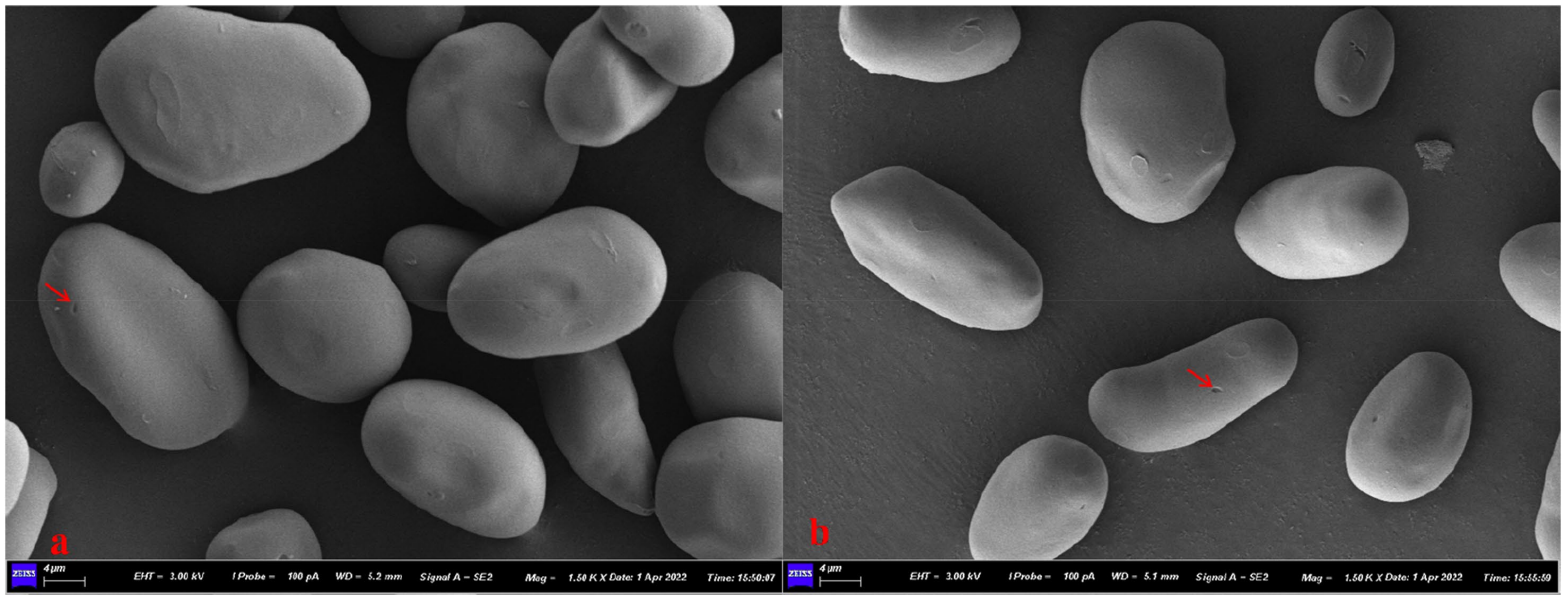
SEM images of bracken starches, Bar = 4 μm: **(A)** Sample J_1_ **(B)** Sample J_2_.

SEM images of bracken starch granules are shown in Figure 1. Most bracken starch granules were near-spherical, ellipsoidal or prolate ellipsoidal, and some were irregular. Pores were observed on surface of some granules, indicated by black arrowheads in Figures 1A,B. According to previous studies (10), it is a natural feature for many sources of starches that the granules contain surface pores. The pore characteristics have profound influence on the starch physicochemical properties and the susceptibility of starch for digestion and modification (11, 12). The above observations were consistent with previous findings in bracken starch (6). Comparing to main-stream starches, bracken starch granules are similar in shape to potato and chickpea starch granules which are mostly oval-shaped, but very different from corn starch granules which are polygonal-shaped (13).

### 3.2. Particle size distribution

Particle size distribution of bracken starch samples were presented in Table 1. D (3,2) and D (4,3) represent area and volume diameters, respectively. D (0.1), D (0.5), and D (0.9) indicate 10, 50 and 90%, respectively, of the starch granules were smaller than the values.

**Table 1:** Structural and physicochemical properties of bracken starches.

Property	Trait	Sample	
		J_1_	J_2_
*Particle size distribution*	D (3,2)/μm	19.4 ± 0.17^a^	16.1 ± 0.05^b^
	D (4,3)/μm	24.5 ± 0.54^a^	18.6 ± 0.15^b^
	D (0.1)/μm	12.8 ± 0.05^a^	10.5 ± 0.06^b^
	D (0.5)/μm	20.4 ± 0.18^a^	17.3 ± 0.09^b^
	D (0.9)/μm	34.1 ± 0.64^a^	28.5 ± 0.48^b^
*Apparent amylose content*	AAC	24.7 ± 0.42^a^	22.6 ± 0.22^b^
*RVA*	PV (cP)	2,859 ± 29.7^a^	3,010 ± 132.9^a^
	HPV (cP)	1,607 ± 15.6^a^	1,593 ± 16.3^a^
	CPV (cP)	2,540 ± 23.3^a^	2,487 ± 17.0^a^
	BD (cP)	1,252 ± 1401^a^	1,416 ± 117^a^
	SB (cP)	-318 ± 6.4^a^	−523 ± 150.0^a^
	CS (cP)	937 ± 7.8^a^	894 ± 33.2^a^
	Ptime (min)	5.8 ± 0.10^b^	6.4 ± 0.09^a^
	PT (°C)	65.1 ± 0.04^a^	65.2 ± 0.03^a^
*Texture properties*	HD (g)	21.3 ± 0.30^a^	18.8 ± 0.27^b^
	ADH (g.s)	−1.24 ± 0.47^a^	−0.25 ± 0.04^a^
	COH	0.786 ± 0.01^a^	0.760 ± 0.003^a^
*DSC*	T_o_ (°C)	59.3 ± 0.06^a^	57.9 ± 0.20^b^
	T_p_ (°C)	63.8 ± 0.11^a^	63.9 ± 0.23^a^
	T_c_ (°C)	69.4 ± 0.28^b^	72.5 ± 0.43^a^
	ΔH_g_ (J/g)	16.7 ± 0.43^a^	15.9 ± 0.04^b^
*XRD*	Relative crystallinity (%)	16.3 ± 0.42^a^	17.7 ± 0.68^a^
*FTIR*	1047/1022	1.57 ± 0.18^a^	1.88 ± 0.05^a^
	1022/995	0.31 ± 0.004^a^	0.35 ± 0.032^a^
*Molecular weight distribution*	Mn (kDa)	21,578 ± 1488^a^	22,997 ± 245^a^
	Mw (kDa)	55,545 ± 3666^a^	54,463 ± 448^a^
	Mw/Mn	2.57 ± 0.008^a^	2.37 ± 0.006^b^
	Rz	166.1 ± 0.90^a^	162.2 ± 0.78^b^
	Branching Degree (%)	4.64 ± 0.26^a^	4.33 ± 0.03^a^
*GPC*	Peak 1 (%)	57.4 ± 0.30^b^	58.9 ± 0.34^a^
	Peak 2 (%)	26.1 ± 0.14^a^	26.4 ± 0.05^a^
	Peak 3 (%)	16.5 ± 0.17^a^	14.8 ± 0.31^b^
*HPEAC*	6<DP ≤ 12	24.7 ± 0.004^a^	24.5 ± 0.232^a^
	12<DP ≤ 24	48.6 ± 0.123^a^	48.8 ± 0.087^a^
	24<DP ≤ 36	12.3 ± 0.007^a^	12.7 ± 0.061^a^
	DP ≥ 37	14.4 ± 0.079^a^	14.0 ± 0.238^a^

All the values are means ± standard deviations. Different letters in the same line indicate significant difference (*p* < 0.05).

AAC, apparent amylose content; ADH, adhesiveness; BD, break down; COH, cohesiveness; CPV, cold paste viscosity; CS, consistency; DP, degree of polymerization; DSC, differential scanning calorimetry; FTIR, fourier transform infrared spectrometer; GPC, gel performance chromatography; GT, gelatinization temperature; HD, hardness; HPEAC, high-performance anion-exchange chromatography; HPV, hot paste viscosity; Mn, number-average molecular weight; Mw, weight-average molecular weight; Ptime, peak time; PT, pasting temperature; PV, peak viscosity; RVA, rapid visco-analyzer; Rz, z-radius of gyration; SB, setback; To, onset temperature; Tc, conclusion temperature; Tp, peak temperature; XRD, X-ray diffraction; ΔHg, enthalpy of gelatinization.

Generally, the bracken starch granules had a mean area diameter no more than 20 μm, and volume diameter less than 25 μm. The J_1_ and J_2_ sample had a mean area diameter of 19.4 μm and 16.1 μm, respectively, this is similar with the previous report which recorded 17.8 μm as the mean diameter for bracken starch granules (6). Basically, the size of bracken starch granules was larger than that of pearl millet, quinoa and amaranth starch granules (14). The size of starch granule is a crucial factor in determining the functional properties and the utilization of starch in food and non-food industries (15).

The two bracken starches varied significantly in D (3,2), D (4,3), D (0.1), D (0.5), and D (0.9) (*p*<0.05). The mean values of all parameters of J_1_ were higher than those of J_2_. This could be attributed to both genetic and environmental factors.

### 3.3. Apparent amylose content

The two bracken starches had AAC of 24.7 and 22.6%, respectively, and AAC significantly different between the two samples (Table 1). In rice, AAC fall into five subgroups: waxy (0–2%), very low (5–12%), low (12–20%), intermediate (20–25%) and high (25–33%) (16). Therefore, bracken starch appeared to have a higher AAC than most of rice cultivars. Amylose content has long been a key indicator of starch quality, and it is positively correlated to gel hardness (17). Generally, starches of high AAC tends to form gel with fluffy and hard texture, whereas low AAC usually leads to a tender and glossy gel. In this respect, it is reasonable that J_1_ sample which had a higher AAC, eventually formed a significantly harder gel after RVA test (Table 1). Apart from gel hardness, AAC greatly affects many other parameters of starch eating and cooking quality. As proposed by Wang et al. (18), AAC, GC and most traits of pasting viscosity can be termed as “AAC-related traits,” since these traits grouped together in hierarchical cluster analysis, and similar findings were reported by other researchers (9, 19).

### 3.4. Pasting properties

The RVA parameters are presented in Table 1. The two samples had very similar RVA patterns, with no significant difference for any parameter, except for Ptime. The starch gelatinization temperatures indexed by PT tested by RVA, were higher than that of indexed by T_p_ tested by DSC (Table 1), which was also noticed by previous studies on other starches (9, 20, 21).

PV, HPV, CPV, BD, SB, CS were 2,859 and 3,010 cP, 1,593 and 1,607 cP, 2,487 and 2,540 cP, 1,252 and 1,416 cP, −523 and − 318 cP, 894 and 937 cP in two bracken starches, respectively. The viscosity of bracken starches appeared to be lower than that of most of rice (21) and potato starch (20). The viscosity values were all much lower than previously reported (6). This could be attributed to many factors. First of all, genetic background can cause huge differences on starch viscosity, this had been well presented in many crops (22–24). Besides, environmental factors can affect starch development and give rise to variation in RVA viscosity (21). Furthermore, different methods and instruments employed by independent studies may generate discrepancy on the values of viscosity parameters. Anyway, the non-negligible difference in the RVA viscosity of bracken starch between studies awaits later studies to investigate.

### 3.5. Gel textural properties

Textural properties were summarized in Table 1. Hardness (HD), Adhesiveness (ADH), Cohesiveness (COH) ranged from 18.8 to 21.3 g, −1.24 to-0.25 g.s, 0.760 to 0.786, respectively. Previous studies have indicated that HD is positively correlated to amylose content (25). HD varied significantly between the two bracken starches, this could be due to the amylose content difference in two samples. Compared to other starches with similar amylose content, bracken starch formed much softer and sticky gel than rice and potato starch (20, 21), and this could be due to the structural properties of starch components. As previously reported, the amylopectin structure also affects the gelation process. The longer chains of amylopectin contribute to a harder texture of starch gel (23, 26). There were no significant differences of ADH and COH between the two samples.

### 3.6. Thermal properties

**Figure 2:**
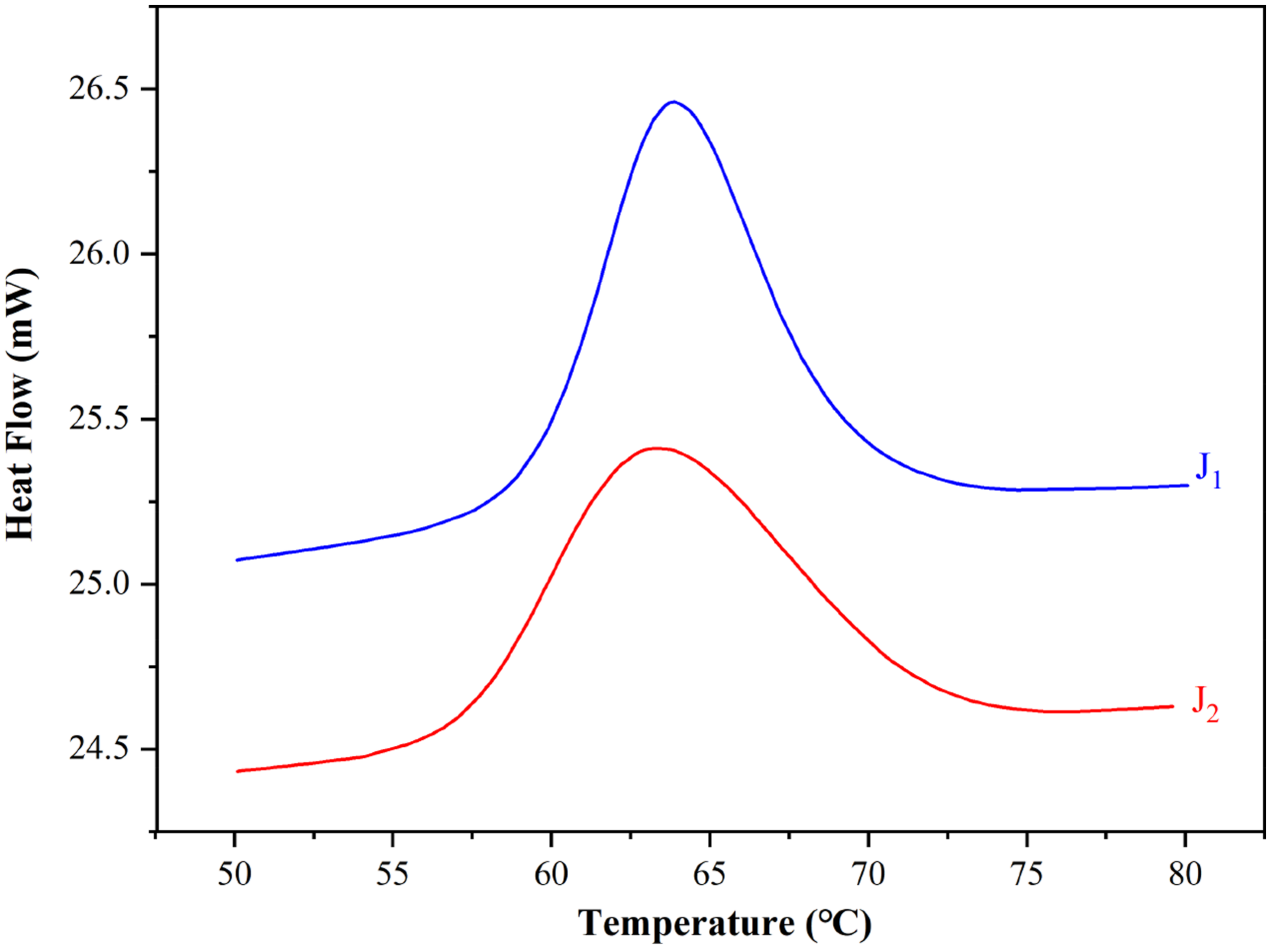
DSC profiles of the two bracken starches.

DSC profiles of the two bracken starches are shown in Figure 2 and gelatinization temperatures and transition enthalpies of bracken starches are summarized in Table 1. For each sample, a single endothermic conversion was observed in the DSC profile (Figure 2). Onset (T_o_), peak (T_p_), conclusion (T_c_) and gelatinization enthalpy (∆H_g_) were 57.9 and 59.3°C, 63.8 and 63.9°C, 69.4 and 72.5°C, and 15.9 and 16.7 J/g, respectively. The two samples varied significantly (*p*<0.05) in all thermal parameters except for T_p_.

Our previous studies found that a set of rice germplasm containing 163 accessions had T_o_, T_p_, and T_c_ ranging from 59.2 to 76.6°C, 66.7 to 81°C, and 71.8 to 87.8°C, respectively (21); the T_o_, T_p_, and T_c_ in 34 foxtail millet accessions ranged from 66.8 to 69.5°C, 72.2 to 73.8°C, and 76.3 to 78.6°C, respectively (9). Other researchers reported that gelatinization temperature (T_p_) varied from 72.5 to 75.7°C in seven maize samples (27), and from 66.4 to 70.2°C in 95 sorghum genotypes (28). Therefore, the gelatinization temperatures of bracken starches were lower than that of many cereal starches. Along with AAC, gelatinization temperature (GT) is the other key indicator of starch quality. GT is the peak temperature in gelatinization process, it reflects the ease or difficulty to cook starch, starch with a high GT needs more energy and time in cooking.

### 3.7. Crystalline structure

There are three types of crystalline structure in starch that can be differentiated by XRD patterns: A, B, and C. A-type is usually found in cereal starch and has peaks at 17°, 18°and 23°while B-type having peaks at 5.5°, 17°, 22°, 24°usually exists in tuber starch. C-type is usually found in bean starches and consists of both A-and B-type (29, 30).

**Figure 3:**
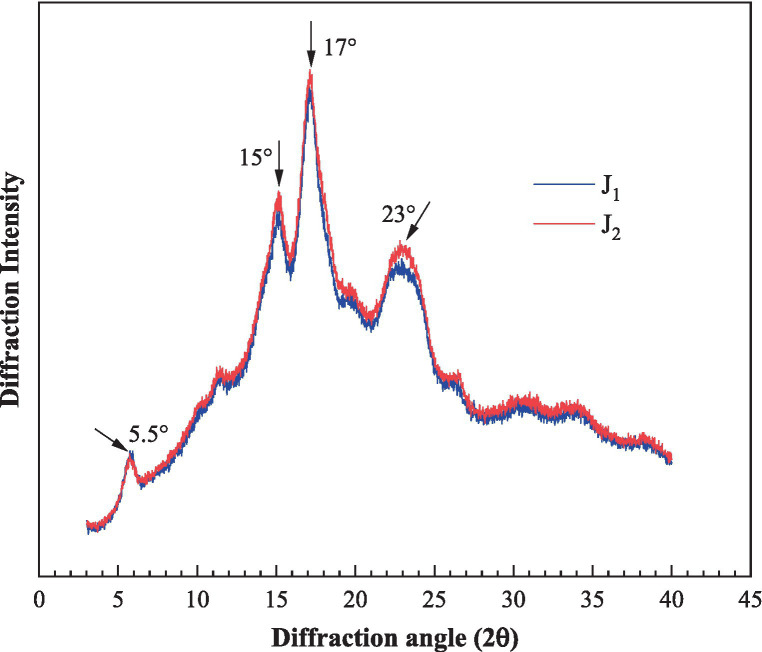
XRD patterns of the bracken starches.

XRD pattern of bracken starches were shown in Figure 3. Peaks of moderate intensity were observed at 5.5°and 15°, followed by a single peak with high intensity appearing at 17°and a broad peak at 23°. This pattern is typical in C-type crystalline structure. The result reported here was in agreement with the previous study of bracken starch (6). The relative crystallinity of J_1_ and J_2_ were 16.3 and 17.7%, respectively, higher than Chinese yam starch but lower than native corn starch (24, 31). The relative crystallinity is an important factor in affecting *in vitro* digestibility of starch, since the relative crystallinity and the resistant starch content are positively correlated, as reported by previous studies (32, 33).

### 3.8. FTIR analysis

**Figure 4:**
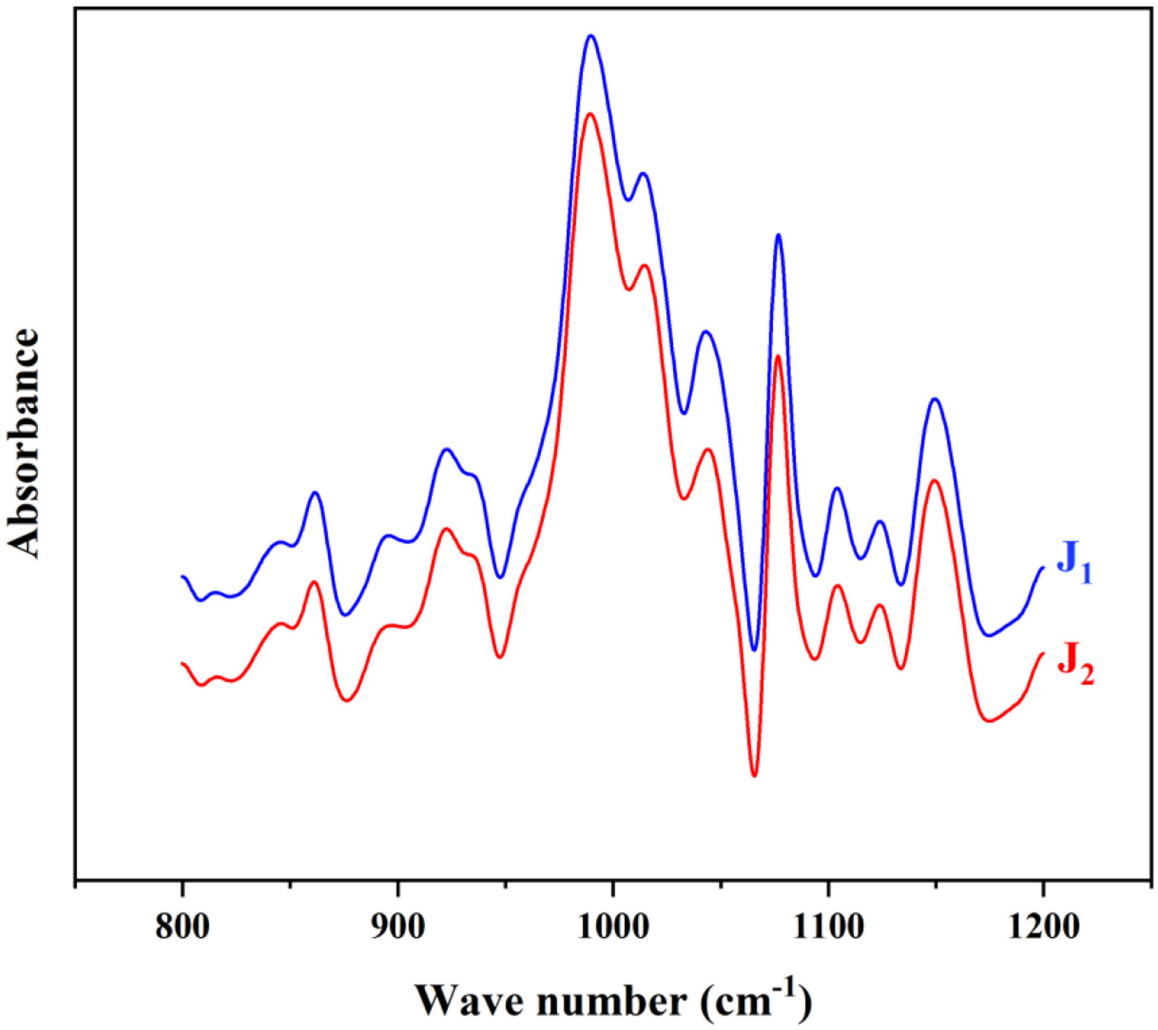
FTIR spectra of the bracken starches.

The short range order of the bracken starches were investigated by FTIR analysis, The absorption peaks at 1047 cm^−1^, 1,022 cm^−1^ and 995 cm^−1^, respectively associated to the crystalline region, amorphous region and hydrated carbohydrate helices of the starch molecule. Therefore, 1,047/1022 ratio indicates the intensity of crystalline and amorphous regions, and 1022/995 ratio indicates the formation of double helix of starch molecules (34). As shown in Table 1 and Figure 4, the two samples had similar absorption peaks, and both 1047/1022 and 1022/995 did not differ significantly between the two bracken starches. Compared to Chinese yam starch, the 1047/1022 of bracken starch was much higher, which suggested a higher crystallinity (24), and this is supported by the data of the relative crystallinity (Table 1). However, both 1047/1022 and 1022/995 had notable differences from the previous report in bracken starch (6), and this could be due to many factors lying in the starch materials and techniques. For example, the moisture content of starch sample can affect the FTIR spectra patterns (35), and different genotypes of *Oryza sativa* (rice) can vary greatly in starch structure (36). Anyway, the exact reason needs to be clarified by later investigations.

### 3.9. Molecular weight distribution

The weight-average molecular weight (Mw), number-average molecular weight (Mn), the degree of the dispersion of the molecular weight distribution (Mw/Mn), and the z-radius of gyration (Rz) of bracken starches were presented in Table 1. Mw, Mn, Mw/Mn, Rz in each sample were 55,545 KDa, 21,578 KDa, 2.57 and 166.1 nm for J_1_, 54,463 KDa, 22,997 KDa, 2.37 and 162.2 nm for J_2_. The two samples varied significantly on Mw/Mn and Rz (*p*<0.05).

The high Mw indicates the starch was composed of highly polymerized amylopectin, and the high ratio of Mw/Mn suggests the molecular weight distribution of starch is highly dispersed (37). The Rz represents the theoretical probability of finding a molecule at a given distance from the centre, higher Rz value means higher branching degree (38). Compared to starches of many other sources, bracken starches showed much higher molecular weight and branching degree, as suggested by Mw, Mn and Rz values (24, 31, 38). The branching degree of J_1_ was slightly higher than that of J_2_, this is in consistent with the variation on Mw, Mn, Mw/Mn, and Rz (Table 1). According to previous studies, the molecular weight of amylose and amylopectin biopolymers can be affected by botanical origin, genotype, and environmental dissimilarities (39, 40).

### 3.10. Relative molecular weight distribution

**Figure 5:**
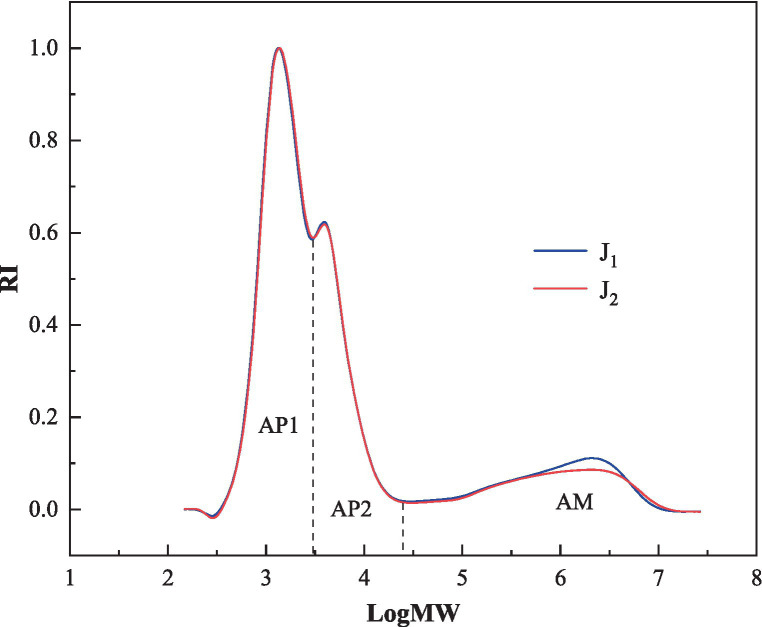
The relative molecular weight distributions of the bracken starches.

Branching characteristics of the isoamylase-debranched bracken starches were determined based on relative molecular weight distribution analysis, using Gel Performance Chromatography (GPC). The results were presented in Figure 5 and Table 1. The three peaks, peak 1, peak 2, and peak 3 in Table 1 represented amylopectin with short-branch chains (AP1), amylopectin with long-branch chains (AP2) and amylose molecules (AM), respectively (41).

The ratios of the three peaks ranged from 57.4 to 58.9%, 26.1 to 26.4%, 14.8 to 16.5%, respectively, in the two bracken starch samples. J_2_ had more proportions of short-branch chains (58.9% *VS* 57.4%) and less amylose chain (14.8 *VS* 16.5) than J_1_ (*p*<0.05). A previous study concluded that higher proportion of amylopectin short chains could lead to higher peak viscosity (PV) and breakdown value (BD), and a softer and stickier texture (HD) of the cooked rice (42). This is also supported by the data of the current study. As shown in Table 1, the bracken starch sample with higher proportion of amylopectin short chains (J_2_) did have higher PV, BD and lower HD values than that of sample with lower proportion of amylopectin short chains (J_1_).

### 3.11. Chain length distribution of the debranched amylopectin

**Figure 6:**
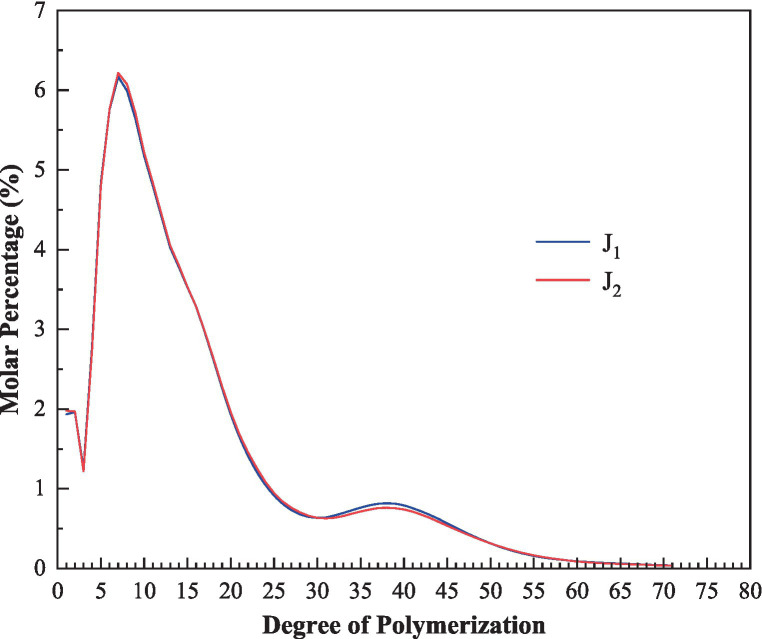
The chain length distribution of debranched amylopectin of the bracken starches.

The chain length distribution of amylopectin was also analyzed by high-performance anion-exchange chromatography-pulsed amperometric detection (HPAEC-PAD). The results were shown in Table 1 and Figure 6. Based on the degree of polymerization (DP) and the model of amylopectin cluster, branched chains are usually classified into four groups: A (DP 6–12), B1 (DP 13–24), B2 (DP 25–36), and B3 (DP ≥ 37) (43, 44).

As shown in Table 1, the proportions of A, B1, B2, and B3 chains of the two samples were 24.7, 48.6, 12.3, 14.4 and 24.5%, 48.8, 12.7, 14.0%, respectively. The bracken starches had similar patterns of amylopectin chain distribution with that of some rice varieties (36, 45). The bracken amylopectin showed higher proportion of B1 chains, lower proportion of B2 and B3 chains than that of Chinese yam (24), but lower proportion of A, B1 chains, much higher proportion of B3 chains than that of corn starch (6). Compared to potato starch, bracken starch had more A and B1 chains, but less B2 and B3 chains (38). There was no significant difference between the two bracken starches in chain length distribution.

## 4. Conclusion

The structural and physicochemical properties of two bracken starch samples were systematically investigated in this study. Most of bracken starch granules were near-spherical, ellipsoidal or prolate ellipsoidal, and had a mean area diameter less than 20 μm. XRD analysis revealed that the bracken starches had typical C-type crystalline structure. During gelatinization event, the bracken starches appeared to have lower viscosity than that of most of rice and potato varieties, and lower gelatinization temperature than cereal starch. Compared to rice and potato starches of similar AAC, bracken starches could form much softer and sticky gels. Therefore, bracken starches have obvious differences with main-steam starches in physicochemical properties, which indicates a potential of utilization for developing food and non-food products. The branch chain length distribution showed that the bracken starches were structurally similar to some rice varieties. There were notable differences in various properties between the two bracken starches. However, the genetic diversity and effect of growing environment on the properties of bracken starch remain to be studied through collection of a large number of samples. This study provides fundamental information for application of bracken starches in food and non-food industries.

## Data availability statement

The raw data supporting the conclusions of this article will be made available by the authors, without undue reservation.

## Author contributions

KL: conceptualization, resources, supervision, data curation, writing-original draft, and writing-review and editing. TZ: writing-original draft, data curation, methodology, and investigation. HR, WZ, and SH: software. YG: methodology. XL: data curation. HC: writing-review and editing and funding acquisition. All authors contributed to the article and approved the submitted version.

## Funding

The authors would like to thank Shantou Science and Technology Bureau (grant no. STKJ2021024), Guizhou University Natural Science Project (2020-23), Guizhou University Seed Program (2020-26), and The Opening Foundation of National Laboratory of Hazard Factors and Risk Prevention of Agricultural Product Quality and Safety (2021DG700024-KF202209) for financial support.

## Conflict of interest

The authors declare that the research was conducted in the absence of any commercial or financial relationships that could be construed as a potential conflict of interest.

## Publisher’s note

All claims expressed in this article are solely those of the authors and do not necessarily represent those of their affiliated organizations, or those of the publisher, the editors and the reviewers. Any product that may be evaluated in this article, or claim that may be made by its manufacturer, is not guaranteed or endorsed by the publisher.
